# Mating induces the expression of immune- and pH-regulatory genes in the utero-vaginal junction containing mucosal sperm-storage tubuli of hens

**DOI:** 10.1530/REP-15-0253

**Published:** 2015-09-08

**Authors:** Mohammad Atikuzzaman, Ratnesh Mehta Bhai, Jesper Fogelholm, Dominic Wright, Heriberto Rodriguez-Martinez

**Affiliations:** Linköping University, Department of Clinical and Experimental Medicine, Faculty of Medicine and Health Sciences, Lasarettsgatan 64/65, Lanken, Floor 12SE-581 85, Linköping, Sweden; Department of Physics, Chemistry and Biology, Faculty of Science and Engineering, Linköping University, Linköping, Sweden

## Abstract

The female chicken, as with other species with internal fertilization, can tolerate the presence of spermatozoa within specialized sperm-storage tubuli (SST) located in the mucosa of the utero-vaginal junction (UVJ) for days or weeks, without eliciting an immune response. To determine if the oviduct alters its gene expression in response to sperm entry, segments from the oviduct (UVJ, uterus, isthmus, magnum and infundibulum) of mated and unmated (control) hens, derived from an advanced inter-cross line between Red Junglefowl and White Leghorn, were explored 24 h after mating using cDNA microarray analysis. Mating shifted the expression of fifteen genes in the UVJ (53.33% immune-modulatory and 20.00% pH-regulatory) and seven genes in the uterus, none of the genes in the latter segment overlapping the former (with the differentially expressed genes themselves being less related to immune-modulatory function). The other oviductal segments did not show any significant changes. These findings suggest sperm deposition causes a shift in expression in the UVJ (containing mucosal SST) and the uterus for genes involved in immune-modulatory and pH-regulatory functions, both relevant for sperm survival in the hen's oviduct.

## Introduction

Following natural mating in chicken, a subpopulation of selected spermatozoa is stored for up to several weeks in the sperm-storage tubuli (SST), the primary sperm reservoir located in the mucosa of the utero-vaginal junction (UVJ) segment of the oviduct (Bakst 2011), while the rest of the ejaculate is voided from the vagina. The SST-stored spermatozoa maintain integrity and potential fertilizing capacity by mechanisms yet unknown. The motility of spermatozoa from chickens, turkeys and quails is inhibited by decreasing the pH level – an effective way to provoke sperm quiescence *in vitro* (Holm *et al*. 1996, Holm & Wishart 1998) and similar to what occurs in the male- (epididymal cauda) and female- (oviduct) sperm reservoirs in mammals (Rodriguez-Martinez 2007). However, whether there are any genes involved in pH regulation *in vivo* has not been investigated. Stored spermatozoa are released from the SST to be present alongside the entire oviduct transported by anti-peristalsis to reach the secondary reservoir in the infundibulum, where fertilization of the ova occurs (Brillard 1993, Bakst 2011, Sasanami *et al*. 2013). Sperm SST-release has been considered a continuous event where aliquots of the stored sperm subpopulation leave the SSTs in relation to various factors, from aquaporin changes in the SST epithelium (Bakst 2011) to ovulation-related progesterone stimuli (Ito *et al*. 2011, Sasanami *et al*. 2013).

Spermatozoa and seminal proteins are antigenic to the female immune system, and should therefore be promptly rejected (Das *et al*. 2009). Moreover, immune-competent cells for acquired immunity, namely macrophages, antigen-presenting cells expressing MHC class II, CD^4+^ and CD^8+^ T cells and premature B and plasma cells have been localized to the mucosal tissue of all avian oviductal segments (Das & Isobe 2008). However, those spermatozoa that survive in the SST seem tolerated by the female during their permanence in the oviduct. In mammals, seminal plasma plays an important role for sperm survival in the female reproductive tract (Robertson 2007) despite its antigenic nature, potentially owing to its immune-modulatory properties that might culminate in a state of functional immune tolerance to paternal antigens (Robertson *et al*. 1997). Such interplay is likely to rely on differential gene expression by the female, either through genes acting on sperm survival or on those involved in immune tolerance. The arrival of spermatozoa to the oviduct leads to changes in its transcriptome or proteome profile as determined in mice (Fazeli *et al*. 2004) and pigs (Georgiou *et al*. 2007). In turkeys, sperm artificial insemination (AI) up-regulated threefold the expression of avidin mRNA in SSTs (Long *et al*. 2003). In chickens, the expressions of transforming growth factor β (TGFβ) and TGFβ- receptors (TBRs) are increased within 24 h after insemination (Das *et al*. 2006). The authors suggested this enhanced gene expression may suppress anti-sperm immune reaction possibly protecting sperm in the SST. In contrast, avian β-defensin, an important factor for innate immunity, is expressed in the mucosa of all oviductal segments; the expression being highest in the vagina and lowest in the SST, suggesting the immune response against pathogens or spermatozoa varies within the tract (Abdel-Mageed *et al*. 2008). A genome-wide gene expression analysis using an oligonucleotide microarray has shown differential expression of transcripts such as Neuropeptide Y, Enah/Vasp-like and of trafficking kinesin-binding protein 1 (responsible for short- and long-term sperm survival) in the SST of Tsaiya Ducks (Huang *et al*. 2011). The mRNA expression of immune-reactive IL1B and LITAF is increased in the vagina up to 6 h after AI in chicken but remains unchanged in the other oviductal segments, indicating that AI can influence the immune reactivity in the vagina but not necessarily in the SST (Das *et al*. 2009). However, information is still lacking as to how the sperm-oviduct interaction post-mating results in sperm survival with intact fertilization potential. We therefore tested the hypothesis that there is a relatively rapid modulatory gene expression shift in the female oviduct that can promote or inhibit their homeostatic action (thereby affecting sperm motility by pH regulation at the UVJ containing mucosal SST) and their immune system to tolerate the presence of allogeneic spermatozoa in the oviduct for lengthy periods. Gene expression changes were explored using cDNA microarray analysis of various segments of the oviduct of hens, comparing un-mated (control) hens to those mated to fertile roosters.

## Materials and methods

### Animals and sources of oviductal segments

The chickens used in this study were derived from an advanced inter-cross line (RJF/WL-L13, 9th generation) between a White Leghorn layer breed (WL-L13, a high egg-laying bird) and Red Junglefowl (RJF, the wild progenitor of the modern chicken with a low laying rate); see Johnsson *et al*. (2012) for details of the cross and breeds used as well for details on rearing and breeding routines. Briefly, all advanced inter-cross line chickens were kept separated by gender at the poultry facilities of Linköping University. Food and water were available ad libitum and the chickens were held under controlled temperature and light regimes (12h light:12h darkness cycle, 5 lux) in 1–2 m^2^ pens depending on age for their first 7 weeks, in compliance with European Community (Directive 2010/63/EU) and Swedish (SJVFS 2012:26) current legislation. Throughout all experiments, animals were handled carefully and in such a way to avoid any unnecessary stress. Semen from sexually mature, proven fertile roosters was collected by manual abdominal massage to confirm their semen quality prior to experimental mating with sexually mature hens. The semen was primarily extended with Dulbecco's medium (1:10 v/v) and examined in four replicates for sperm concentration and kinematics using a light microscope equipped with a thermal plate (41 °C), positive phase contrast optics (10× objective), a Charge Coupled Device (CCD) camera (UI-1540LE-M-HQ, IDS Imaging Development Systems, Obersulm, Germany), and the Qualisperm Software (Biophos SA, Lausanne, Switzerland). To comply with the optimal functioning of the software algorithm, the extended semen was further extended with the same medium to a final 1:250 rate. Hens (*n*=8) were mated and, 24 h later, euthanized by cervical dislocation and decapitation, along with unmated hens (controls, *n*=4). Both mated and control hens were maintained in the same husbandry conditions, following Swedish regulations, as previously described. Immediately post-mortem, the female oviduct was dissected out, and segments of the oviduct identified (UVJ, uterus, isthmus, magnum and infundibulum) under stereomicroscopy. Representative transversal samples were then collected at every segment, in its mid-region, following classical descriptions (Bakst 1998) and snap-frozen in liquid nitrogen (LN_2_), prior to being stored at −80 °C until being processed. Collection instruments, gloves and specimen holders were changed between each specimen to avoid confounding contamination. A supplementary UVJ sample was obtained from each mated hen and fixed in 4% formaldehyde for histological confirmation of sperm presence in the SST-reservoirs.

### Ethics statement

The experiments were approved well in advance by the ‘Regional Committee for Ethical Approval of Animal Experiments’ (Linköpings Djurförsöksetiska nämnd) in Linköping, Sweden (permit no 75–12).

### cDNA microarray

A total of 36 microarrays were run for this experiment. In the case of UVJ-segments, four control females and eight mated females were used (12 arrays). In the case of the remaining oviductal segments (uterus, isthmus, magnum and infundibulum), three control and three mated females were used (24 arrays). Total RNA was extracted from the various samples using TRIzol (Invitrogen). Total RNA from each sample was quantified using a NanoDrop 1000 (Thermo Fisher Scientific, Fremont, CA, USA), with RNA integrity (RIN ≥8) assessed using an Agilent 2100 Bioanalyzer (Agilent Technologies, Inc., Santa Clara, CA, USA). Double stranded cDNA was synthesized using RevertAid Premium First-Strand cDNA Synthesis Kit (Thermo Fisher Scientific) following the manufacturer's instructions. The ds-cDNA samples were cleaned, labeled, hybridized and washed according to the manufacturer's protocols of the Roche Nimblegen 12×135 k arrays, described elsewhere (Li *et al*. 2013) and the manufacturers guidelines as detailed in the Gene Expression Analysis protocol (Roche NimbleGen Systems, Inc.). The cDNA microarray used custom-designed 12×135 k array slides for samples of UVJ, uterus, isthmus, magnum and infundibulum, derived from control (unmated) and mated birds. The array included all Ensembl (Flicek *et al*. 2012) and RefSeq (Pruitt *et al*. 2009) chicken transcripts. As well as all known transcripts, the array included probe sequences from a chicken brain cDNA library (Boardman *et al*. 2002), which provided a further 10 686 probesets. Three 60-mer-oligonucleotide probes represented each transcript. To avoid SNPs in probe sequences, all known SNP position derived from the recent resequencing of Red Junglefowl and domestic chickens (Rubin *et al*. 2010) were masked, so that probes could not be chosen from sequences with known SNPs. This array design has been utilised extensively in previous work with the chicken strain utilised in this study (an advanced inter-cross between Red Junglefowl and White Leghorn birds). A targeted expression QTL analysis that utilised the comb tissue from 39 males (i.e. 39 arrays were used in the study) (Johnsson *et al*. 2014) was found to corroborate and develop the results previously obtained using qPCR (Johnsson *et al*. 2012). Furthermore, this same array design has been successfully used in a separate expression QTL study, this time involving the hypothalamus tissue of 129 advanced inter-cross individuals (Johnsson *et al*. 2015). Given these extensive studies using a variety of tissue types in the identical strain to that used in this study, and the custom nature of the design of this microarray, we have strong support for its reliability and the replication of results. The current microarray data are available in the ArrayExpress database (www.ebi.ac.uk/arrayexpress) (Kolesnikov *et al*. 2015) under the accession number E-MTAB-3327.

### Quantitative PCR assay

The tissue samples used for the qPCR experiments were the same samples used for the microarray experiments. Verification qPCRs were performed for four of the differentially expressed genes detected (for primer details see Supplementary Table 1, see section on supplementary data given at the end of this article) in the UVJ containing mucosal SST. First strand cDNA for qPCR was made with Fermentas (St Leon-Rot, Baden-Württemberg, Germany) RevertAid Reverse Transcriptase, using 10 mM dNTPs, RiboLock nuclease inhibitor, and oligo(dT)_18_ primer (Thermo Fisher Scientific), according to the manufacturer's protocol. qPCR was performed with Maxima SYBR Green qPCR mastermix (Thermo Fischer Scientific) in 15 μl reactions with 0.3 M of each oligonucleotide primer on a Rotor-Gene 6000 real-time cycler (Corbett Research, Cambridge, UK). The PCR program consisted of a 10 min activation step at 95 °C, followed by 40 cycles of 15 s at 95 °C, and 1 min at 60 °C. After cycling, products were melted by ramping the temperature from 72 °C to 95 °C. The qPCR data was analysed with the comparative ΔΔCt method (Livak & Schmittgen 2001). The qPCR has been run in triplicate per gene per sample. Average Ct value of three housekeeping genes (reference genes)- β2 microglobulin, TATA box binding protein, and RNA polymerase II subunit C1- was subtracted from the average Ct value of target gene (control, mated) to calculate ΔCt of target gene. Normalized target gene expression in mated hens was calculated by a formula 2^(−ΔΔCt)^.

### Statistical analysis

Semen variables (sperm concentration and motility) are expressed as mean±s.e.m. Data were analysed using a non-parametric *t*-test (SPSS IBM corp. 2012 version 21). For the microarray, the slide was scanned following the protocol for scanning one-color NimbleGen arrays with the MS 200 Microarray Scanner and the MS 200 Data Collection Software. Scanned images (TIFF format) were then imported into DEVA Software (Roche NimbleGen, Inc, DEVA 1.2.1) for grid alignment and expression data analysis. Expression data were normalized through quantile normalization and the Robust Multichip Average (RMA) algorithm included in the Deva Software. Statistical analysis of normalized gene expression data was carried out using open source R (Version 3.1.2) software package. Dimensionality reduction was obtained through Principal Component Analysis (PCA) using package ‘FactoMineR’ and plotted using ‘ggplot2’ along the first two principal component capturing most of the variation in the data. Linear model using the empirical Bayes' approach as implemented in the package ‘limma’ was used to calculate differentially expressed genes in all oviductal segments between control (*n*=3) and mated females (*n*=3) except UVJ, where four controls and eight mated individuals were used. Multiple testing was carried out using False Discovery Rate (FDR) and 5% FDR significance threshold (equivalent to a *P* value of 0.05) was used to declare a significant difference (Adjusted *P* value, *q*) between populations.

Gene ontological (GO) classification and functional analysis was carried out using an open source Panther Classification System (http://pantherdb.org) (Mi *et al*. 2013) and UniProtKB (http://www.uniprot.org/) (Magrane & Uniprot 2011). The GO classified data were then exported into Microsoft Excel 2013 to produce pie chart figures for GO categories.

## Results

**Figure 1:**
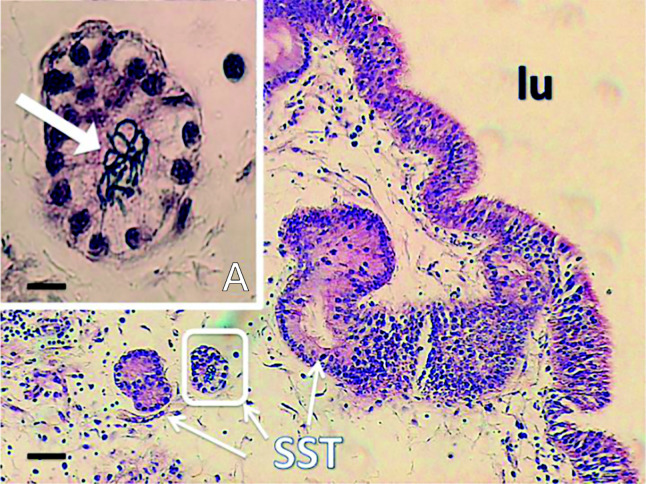
Representative histology of the UVJ containing mucosal SST holding the sperm. Microphotograph of a section of the UVJ of a mated hen (24 h post-mating) depicting sections of SST, Bar: 100 μm, HE. In (A) a higher magnification (Bar: 10 μm) of a marked SST depicts spermatozoa in the lumen (thick arrow), Lu: lumen of the UVJ.

The semen of the roosters used in the experiment varied in sperm concentration (1.2±0.6–5.9±0.7 billion/ml, mean±s.e.m.) and sperm progressive motility (74.4±15.8–99.00±0.6%), within ranges reported for RJF and commercial layers (Malik *et al*. 2013). All mated hens had spermatozoa in their SST, as representatively depicted in Fig. 1.

### Differential gene expression between mated and control individuals in the UVJ containing mucosal SST and uterus

**Figure 2:**
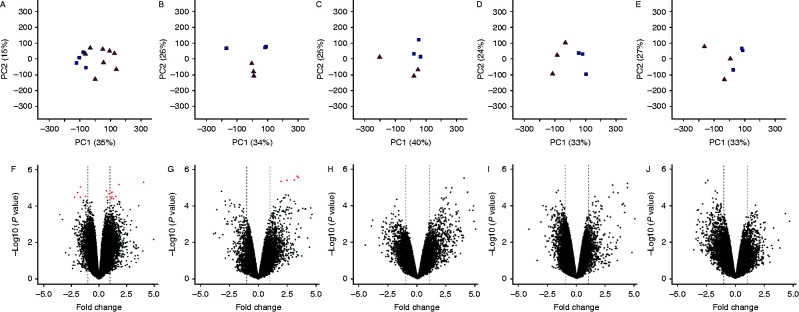
PCA and volcano plots for gene expressions in all oviductal segments (control vs mated individuals). First and second significant components (A, B, C, D and E) show 50, 60, 65, 57 and 60% variation in UVJ, uterus, isthmus, magnum and infundibulum respectively. Volcano plots (F, G, H, I and J) show up- and down-regulated gene expressions in UVJ, uterus, isthmus, magnum and infundibulum. The *X*-axis represents fold change (FC) in gene expression and *Y*-axis represents –log10 of *P* value. Vertical dashed lines represents logFC cut off values −1.00 or +1.00 and red dots represent differentially expressed genes (*q*≤0.05).

The cDNA microarray revealed differential gene expression in the UVJ containing mucosal SST and the uterus while the isthmus, magnum and infundibulum remained unchanged between control and mated individuals (Fig. 2). Analysis of the first and second components of the PCA (Fig. 2A, B, C, D and E) showed 50–65% of the total variation came from between-groups despite volcano plots (Fig. 2F, G, H, I and J) indicating only the UVJ and the uterus showed a significant differential expression of certain genes. In total, 15 genes were differentially regulated between control and mated birds in the UVJ containing mucosal SST, and seven in the uterus (see Table 1).

### Classification of differentially expressed genes in UVJ containing mucosal SST and uterus in response to mating

**Figure 3:**
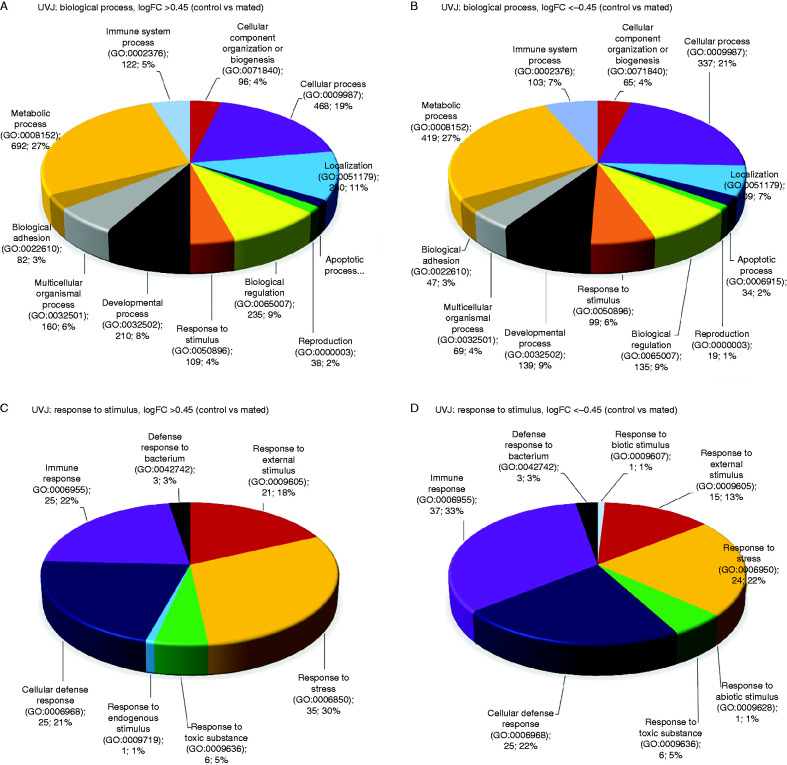
Gene ontology (GO) analysis of differentially expressed (logFC either >0.45 or <−0.45) genes in UVJ (control vs mated individuals). Data label represents category name (Accession), number of genes and percent of gene hit against total number of biological process hits. (A) Total number of genes (*n*)=1539; total number of biological process hits (*N*)=2532. (B) *n*=1120; *N*=1575. (C) *n*=109; *N*=116. (D) *n*=99; *N*=112.

Differentially expressed genes (control vs mated) (*q*≤0.05) have been classified for functionality based on both online database services and peer reviewed published articles (Table 2). The highest (eight) and second highest (three) number of differentially expressed genes in the UVJ containing mucosal SST were found classified as immune regulatory (53.33%) and pH-regulatory (20%) respectively. In the case of the uterus, there was no particular enrichment of any one category, though the number of differentially expressed genes was so small this is hardly surprising. Additional gene ontology (GO) analyses results of differentially expressed genes based on log fold change with a lower significance threshold (for up regulated genes, logFC >0.45 and for downregulated genes, logFC <−0.45) are shown in Fig. 3 for the UVJ and Supplementary Figure 1, see section on supplementary data given at the end of this article for the remainder of the oviductal segments. Twelve categories were identified (see Fig. 3), with metabolic process (GO: 0008152) consistently being the largest GO category identified in each sample tissue. However, the immune system process (GO: 0002376) and response to stimulus (GO: 0050896) categories were also identified (Fig. 3). The immune system process is directly related to sperm survival and it was therefore considered to be of prime interest for further investigation. The GO term ‘response to stimulus’ was also considered important for further investigation, because gene shifts in this category might be due to the stimuli produced by the post-mating spermatozoa in the UVJ (Fig. 1). A total of 122 up-regulated and 103 down-regulated genes in the UVJ of mated hens were categorized as being related to the immune system process (Supplementary Table 2). Similarly, 109 up-regulated and 99 down-regulated genes were found in the GO term category ‘response to stimulus’ (Supplementary Table 3). The possible roles of these up- and down-regulated genes in the UVJ have been summarized in Supplementary Tables 2 and 3.

### Differential gene expression by oviductal segments

A comparison of expression between oviductal segments for down- and up-regulated genes is presented in Table 3. Irrespective of mating, the UVJ containing mucosal SST possessed the greatest number of down-regulated genes, as compared to the other segments. The UVJ, uterus and magnum had greater numbers of up-regulated genes. In terms of the number of unique (specific to a single segment) genes that were suggestively differentially expressed (logFC >0.45 or logFC <−0.45) in the UVJ, we found a total of 1712 genes were up-regulated and 977 genes were down-regulated (see Supplementary Excel file 1, see section on supplementary data given at the end of this article, where the further gene ontology analysis results of these genes can be seen).

### Four differentially expressed genes in the UVJ were validated by qPCR

**Figure 4:**
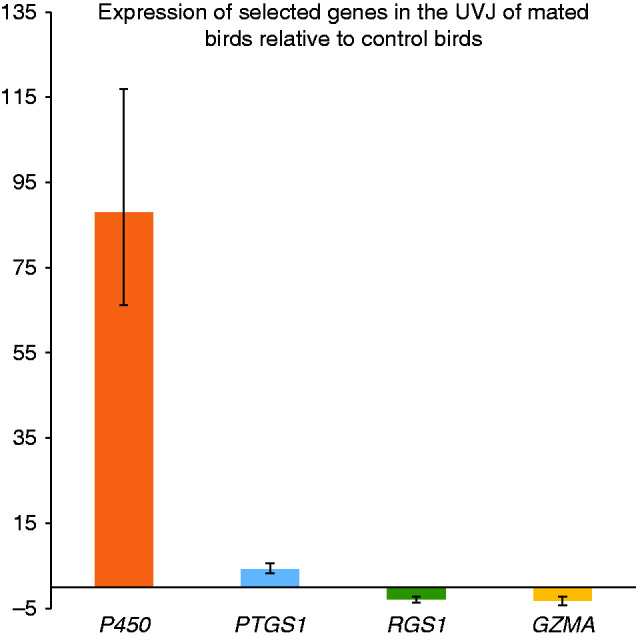
Quantitative PCR verification of microarray results. Two up-regulated (logFC >0.45) genes- P450, PTGS1 and two down-regulated (logFC <−0.45) genes- RGS1, GZMA have been verified using qPCR assay. The *Y*-axis represents mean expression (calculated by 2^−ΔΔCt^) of genes and the error bars represents ±s.e.m.

The two up-regulated (logFC >0.45) genes (P450 and PTGS1) and the two down-regulated (logFC <−0.45) genes (RGS1 and GZMA) revealed by microarray analysis were also expressed in the same direction when measured by real time quantitative polymerase chain reaction (Fig. 4), to verify the microarray results (see also methods section for further confirmation analysis).

## Discussion

The present study reveals that sperm deposition during natural mating causes relatively rapid (within 24 h) changes in the expression of genes involved in immune-modulatory and pH-regulatory functions, both relevant for sperm survival in the reproductive tract of hens. However, these changes are apparent only in the UVJ containing mucosal SSTs and the uterus. Absence of such significant gene expression shifts in other areas, indicates that the UVJ function requires up- or down-regulation of specific genes within a brief period post-entry, to warrant the storage of sufficient fertile spermatozoa for fertilization in the primary sperm reservoir (mucosal SSTs).

The examination of the UVJ revealed that both immune-reactive and immune-suppressive genes were differentially expressed in mated hens. The immune-modulatory genes found in the current study have also been related to immune regulation by other studies. For instance LMBRD2- is responsible for cellular migration in Dictyostelium dicoideum (Kelsey *et al*. 2012); GZMA – is able to produce local inflammatory response in the target cells (Irmler *et al*. 1995, Catalfamo & Henkart 2003, Bots & Medema 2006); PDE7A is expressed in human T cells (Mary *et al*. 1987, Krause & Deutsch 1991, Soderling & Beavo 2000); RGS1 is a regulator of G protein-couple receptors (GPCR) (see review Cho & Kehrl (2009)); PLCH1 is responsible for GPCR mediated signaling in mouse neuroblastoma cells Neuro2A (N2A) cells (Kim *et al*. 2011) and CPAMD8 in human is also related to immune-regulation (Philip *et al*. 1994, Volanakis 2002, Skornicka *et al*. 2004, Athippozhy *et al*. 2011, Jeng *et al*. 2011). Moreover, the up-regulation of P450 and PTGS1 in the UVJ of mated hens could potentially indicate the synthesis of prostaglandin in this area. PTGS-derived prostaglandins are involved both in oviductal motility (Brillard 1993) as well as in immune-modulation (Harris *et al*. 2002, Nebert & Dalton 2006). Following reports that spermatozoa could stimulate prostaglandin synthesis in bovine oviductal cells (Kodithuwakku *et al*. 2007), we hereby speculate that the entry of spermatozoa and the over-expression of PLA2G2E in the UVJ-area might enhance the up-regulation of PTGS1 and prostaglandin synthesis (Murakami *et al*. 2002). Interestingly, the gene P450 has been indicated as being differentially expressed in Drosophila melanogaster females in response to mating (McGraw *et al*. 2004).

One could argue that male courtship and sexual harassment of the females, or even mating could have influenced the females and their oviducts, rather than – or concerted with – the presence of sperm or seminal fluid. Hormonal and gene induction changes at brain level are elicited by these events (Ball & Balthazart 2001) but evidence of changes at the oviduct level is yet, to the best of our knowledge, not available. Use of artificial insemination could provide some cues, by waiving this eventual male courtship/mating factor.

Spermatozoa are sensitive to pH and their motility is rapidly affected by changes in pH levels. In domestic mammals (cow and pigs) and avian (chicken, quail and turkey) *in vitro* sperm motility is highest at an alkaline pH and can be manipulated towards quiescence by exposure to low pH (Holm & Wishart 1998, Rodriguez-Martinez 2007). In chicken, pH values below 7.8 inhibit sperm motility, and at this level sperm motility remains low, while raising the pH value 0.2 units and higher provides vigorous sperm motility (Holm & Wishart 1998). *In vivo*, porcine spermatozoa are quiescent in the cauda epididymides (pH 6.5, Rodriguez-Martinez *et al*. 1990); motility becoming activated by exposure to high pH or increasing bicarbonate levels (Rodriguez-Martinez 2007). Interestingly, the oviductal sperm reservoirs of the sow register lower pH levels (6.7) compared to the upper tubal segments where fertilization takes place (ampullary-isthmic junction: 7.5; ampullae: 8.3) (Rodriguez-Martinez 2007) adding circumstantial evidence to suggestions that changes in pH from acidic to alkaline would also regulate sperm transfer to the fertilization site (Holm *et al*. 1996). Interestingly, our current results indicate that the entry of spermatozoa to the SST at UVJ causes alterations in the expression of pH-regulatory genes such as ATP13A3, SLC12A8, and RHAG. ATP13A3 potentially regulates pH by ion (Na^+^ or K^+^) and proton (H^+^) exchange between intra and extracellular spaces (Pang *et al*. 2001, 2004, Bublitz *et al*. 2011, Palmgren & Nissen 2011). Similarly, SLC12A8 also affects ion exchange (Arroyo *et al*. 2013), whilst RHAG functions in the exchange of protons between intra and extracellular spaces (Westhoff *et al*. 2004, Benjelloun *et al*. 2005). Therefore, it is possible that variation in pH is related to sperm quiescence during storage in the SST. Further studies are obviously needed to explore pH in the SST.

The unique nature of the UVJ containing mucosal SST is also revealed by the large gene expression shifts that are unique to this segment at all times (irrespective of whether mating has taken place or otherwise). The UVJ had the greatest number of down-regulated genes relative to the other segments of the oviduct in the control birds, potentially preparing the area for the presence of foreign spermatozoa. Post insemination, the UVJ showed once again the greatest number of down-regulated genes relative to the other oviductal segments (Table 3). Spermatozoa are retained in the SST for a longer duration than in any of the other compartments due to the nature of avian reproduction, making this compartment essential for sperm survival. Gene ontology (GO) analysis of the differentially expressed genes in the UVJ showed an enrichment of 12 gene classes, among them several involved in the orchestration of immune-regulation (GO: 0002376: immune system; GO: 0050896: the response to stimulus) (Fig. 3A and B). Up to 122 up-regulated and 103 down-regulated genes were involved in immune system processes. Interestingly, most of the down-regulated genes in the immune system process category belong to the immune reactive functions while up-regulated genes in this category belong to immune reactive, immune suppressive and other functions (see Supplementary Table 2, see section on supplementary data given at the end of this article). The presence of spermatozoa in the SST at UVJ confirmed that this compartment was colonized in mated females. GO analysis revealed the majority of the down-regulated genes in the GO term category ‘response to stimulus’ are immune responsive while the majority of the up-regulated genes are related to stress responsiveness (Fig. 3C and D). The data indicate that immune responsive genes are down regulated in the UVJ of mated hens, which might favor the survival of spermatozoa in its mucosal SSTs.

Of the seven genes up-regulated in the uterus of mated hens, Ovocleidin 116 is a candidate molecule for the regulation of calcite growth during egg shell calcification (Hincke *et al*. 1999). GKN2 is an eggshell specific protein (Jonchère *et al*. 2010) while the function of KNG1 is yet unknown. IGFN1 is responsive to stress and elasticity (Erickson 1994, Mansilla *et al*. 2008). KCNV1 is a voltage-dependent K^+^ exchanger (González *et al*. 2012). ADORA2A is a G-protein-coupled receptor partly responsible for the immune modulatory pathway (Haskó *et al*. 2000). Cyp51A1, which is a member of cytochrome P450 family, is responsible for prostaglandin synthesis (Nebert & Dalton 2006), and appears to help in the survival of pre-fertilized spermatozoa as well as aids in egg shell formation and increasing elasticity in the uterus (Table 2). The Ovocleidin 116 and GKN2 are also reported to be up-regulated in the uterus of laying hens when compared to juvenile hens (Dunn *et al*. 2009).

Summarizing all the differentially expressed genes, we can speculate that changes in the expression of genes in the UVJ containing mucosal SST of mated hens might participate in immune-modulation and the regulation of pH in the segment. Changes in the expression of genes in the uterus might be involved in egg shell formation and immune-modulation while gene expression shift in other segments of the oviduct remained non-significant between control and mated hens. Such immune-modulatory and pH-regulatory gene shifts in the UVJ could promote sperm survival by immune-suppression while immune-reactivity might eliminate dead spermatozoa or sperm/seminal fluid debris. Similarly, changes in local pH might keep spermatozoa quiescent or increase their motility depending on whether they will be retained in the SST or released from this compartment for fertilization. However, further research is required to explore the roles of each of the differentially expressed genes regarding cross-talk between spermatozoa and the oviduct of mated chicken.

## Supplementary data

This is linked to the online version of the paper at http://dx.doi.org/10.1530/REP-15-0253.

## Figures and Tables

**Table 1:** Differentially expressed genes in the UVJ containing mucosal SST and the uterus of mated hens compared to unmated (control) hens at 5% FDR corrected *P* value (*q*≤0.05).

**Gene symbol or ID**	**Tissue**	**ENSEMBL/UniProtKB ID**	**Gene name**	**logFC**	***P* value**	***q* value**
Up-regulated						
LOC424944	UVJ	ENSGALG00000008283/F1P1T3	Cytochrome P450 2J2-like (LOC424944)	4.04	4.90×10^−06^	0.047
*PLCH1*	UVJ	ENSGALG00000010312/E1C7E3	Phoshpolipase C *et al*.	1.82	6.46×10^−06^	0.047
*RHAG*	UVJ	ENSGALG00000016684/F1NFG6	Rh-associated glycoprotein	1.52	2.94×10^−05^	0.048
*PLA2G2E*	UVJ	ENSGALG00000014176/F1NZ96	Phospholipase A2, group IIE	1.19	1.73×10^−05^	0.048
*CPAMD8*	UVJ	ENSGALG00000003742/F1NN85	C3 and PZP-like, alpha-2-macroglobulin domain containing 8	1.17	3.05×10^−05^	0.048
*ATP13A3*	UVJ	ENSGALG00000007075/E1C7N6	ATPase type 13A3	1.15	1.84×10^−05^	0.048
*C17ORF85*	UVJ	ENSGALG00000002653/F1NGX2	Chromosome 19 open reading frame, human C17orf85	1.11	3.78×10^−05^	0.049
*SLC12A8*	UVJ	ENSGALG00000012045/F1NG01	Solute carrier family 12, member 8	0.93	2.09×10^−05^	0.048
LOC771318	UVJ	ENSGALG00000015516/F1NR26	Phosphodiesterase 7A	0.72	3.16×10^−05^	0.048
*LMBRD2*	UVJ	ENSGALG00000013377/E1BV17	LMBR1 domain containing 2	0.67	1.49×10^−05^	0.048
*GKN2*	Uterus	ENSGALG00000000119/E1C2G7	Gastrokine 2	8.40	1.71×10^−06^	0.019
LOC395256	Uterus	ENSGALG00000010927/F1NSM7	Matrix extracellular phosphoglycoprotein	7.79	9.04×10^−09^	0.0001
*IGFN1*	Uterus	ENSGALG00000000295/E1C7I7	Immunoglobulin-like and fibronectin type III domain containing 1	3.46	4.95×10^−06^	0.024
Q7LZS0_CHICK	Uterus	ENSGALG00000008678/E1BX43	Kininogen 1	3.36	4.21×10^−06^	0.024
*KCNV1*	Uterus	ENSGALG00000016109/E1BQJ2	Potassium channel, subfamily V, member 1	3.12	6.63×10^−06^	0.024
*ADORA2A*	Uterus	ENSGALG00000006642/E1BXP5	Adenosine receptor A2	2.48	6.78×10^−06^	0.024
Q0KKP4_CHICK	Uterus	ENSGALG00000009365/F1P0L8	Cytochrome P450, family 51, subfamily A, polypeptide 1	1.98	7.79×10^−06^	0.024
Down-regulated						
*RGS1*	UVJ	ENSGALG00000002549/E1BU64	Regulator of G-protein signaling 1	−2.20	3.27×10^−05^	0.048
*GZMA*	UVJ	ENSGALG00000013548/F1N917	Granzyme A (granzyme 1, cytotoxic T-lymphocyte-associated serine esterase 3)	−1.92	1.80×10^−05^	0.048
LOC417962	UVJ	ENSGALG00000011799/E1BQK1	Uncharacterised (LOC4179620)	−1.68	3.32×10^−05^	0.048
*FGF18*	UVJ	ENSGALG00000002203/Q9I950	Fibroblast growth factor 18	−1.59	7.43×10^−07^	0.016
ENSGALG00000013955	UVJ	ENSGALG00000013955/1BW70	Uncharacterised	−1.21	2.89×10^−05^	0.048

**Table 2:** Functional classification of differentially expressed (control vs mated) genes at 5% FDR corrected *P* value (*q*≤0.05) in the UVJ containing mucosal SST and uterus of hens.

**Category**	**Gene symbols**	**Number of genes (%)**	**Tissue**
Immune-modulatory	*LMBRD2, CPAMD8, P450, PLA2G2E, RGS1, PDE7A, GZMA, PLCH1*	8 (53.33)	UVJ
pH-regulatory	*ATP13A3, SLC12A8, RHAG*	3 (20)	UVJ
Growth factor	*FGF18*	1 (6.67)	UVJ
Uncharacterized	*C17orf85, LOC417962*, ENSGALG00000013955	3 (20)	UVJ
Receptor activity	ADORA2A	1 (14.29)	Uterus
Structural molecule activity	*IGFN1*	1 (14.29)	Uterus
Transporter activity	*KCNV1*	1 (14.29)	Uterus
Egg shell formation related	*Ovocleidin 116, GKN2*	2 (28.51)	Uterus
Unknown	*KNG1*, Q0KKP4_CHICK	2 (28.51)	Uterus

**Table 3:** Inter-segmental differential gene expressions in control and mated hens.

		**Total downregulated genes**				
		Infundibulum	Magnum	Isthmus	Uterus	UVJ
Infundibulum	Control		2586	1255	1399	4630
	Mated		3281	714	951	1381
Magnum	Control	2328		875	1195	4592
	Mated	2588		456	1361	3152
Isthmus	Control	8351	7391		596	7313
	Mated	5409	5827		1309	5117
Uterus	Control	7649	6806	308		5945
	Mated	7177	8733	1100		5084
UVJ	Control	7902	7389	2868	2653	
	Mated	5694	7523	2564	1814	
		**Total upregulated genes**				
